# How education systems shape cross-national ethnic inequality in math competence scores: Moving beyond mean differences

**DOI:** 10.1371/journal.pone.0193738

**Published:** 2018-03-01

**Authors:** Christoph Spörlein, Elmar Schlueter

**Affiliations:** 1 Chair of Sociology and the Analysis of Social Structures, Otto-Friedrich-University, Bamberg, Germany; 2 Institute of Sociology, Justus Liebig University, Giessen, Germany; University of Westminster, UNITED KINGDOM

## Abstract

Here we examine a conceptualization of immigrant assimilation that is based on the more general notion that distributional differences erode across generations. We explore this idea by reinvestigating the efficiency-equality trade-off hypothesis, which posits that stratified education systems educate students more efficiently at the cost of increasing inequality in overall levels of competence. In the context of ethnic inequality in math achievement, this study explores the extent to which an education system’s characteristics are associated with ethnic inequality in terms of both the group means and group variances in achievement. Based on data from the 2012 PISA and mixed-effect location scale models, our analyses revealed two effects: on average, minority students had lower math scores than majority students, and minority students’ scores were more concentrated at the lower end of the distribution. However, the ethnic inequality in the distribution of scores declined across generations. We did not find compelling evidence that stratified education systems increase mean differences in competency between minority and majority students. However, our analyses revealed that in countries with early educational tracking, minority students’ math scores tended to cluster at the lower end of the distribution, regardless of compositional and school differences between majority and minority students.

## Introduction

Social science research has long established that upon arrival in the host society immigrants often occupy disadvantaged socioeconomic positions in comparison to the majority group. While many theoretical lenses have proven useful for approaching the subsequent multifaceted process of immigrant integration [1–5], one persisting question is this: why do the gaps in achievement between majority and minority groups show considerable variation between immigrant groups and across receiving contexts? Research on this issue has increased in momentum and complexity in recent decades, because the long-term integration of immigrants into host societies has been on the public agenda since mass immigration became a common feature of Western societies [6–7].

Moreover, in response to global trends, the social sciences have acquired the necessary research tools and the empirical sources (e.g., large-scale cross-national comparative datasets) to study differences in immigrant integration across destination countries and origin groups. Core dimensions of integration include language acquisition, educational attainment, labor market outcomes, intimate interethnic relationships, and religiosity, but also immigrants’ social identification or perceptions of discrimination, to name just a few examples [8–17]. Despite this apparent progress in understanding ethnic inequality, there is always room to increase the complexity of conceptual models to generate deeper insights.

One crucial way of achieving this is to move beyond the social sciences’ current focus on mean differences. Instead, social science researchers should move toward a more general framework of distributional inequality that examines intergroup inequality in terms of both group mean differences and conditional variance around the respective group means [18]. This is not a matter of merely adding another orthogonal dimension of inequality to mean differentials. Rather, this involves the study of a relatively neglected but important aspect that suggests the potential for group-specific generational mobility or the lack thereof (i.e., entrenchment of ethnic stratification). Take, for example, the educational achievement of two minority groups in comparison to the majority population. Differences in the minority groups’ educational achievement that persist even after relevant compositional differences (e.g. in terms socioeconomic resources) between groups have been taken into account are commonly interpreted as resulting from anti-minority discrimination and subsumed under the term ethnic penalty [13]. However, whereas the educational achievement distribution of one group is tightly clustered around the lower end of the distribution, the second group may be less concentrated and the distribution of their educational achievement may be wider. Thus, the second group has higher *ethnic mobility potential*, in that they likely share more interaction contexts with the majority group and may be on a faster path toward socioeconomic integration [19]. From a policy perspective, a higher ethnic mobility potential may also be indicative of less inequality in educational opportunity between majority and minority students. Examining group differences in terms of concentration or variation around mean differentials is fairly straightforward: We simply need to add new methodologies to our common set of research tools, as information on variance differences is already contained in virtually all (quantitative) social science data. Moreover, the case for investigating group differences in variance from a substantive point of view rather than treating it as a statistical nuisance is made frequently across (social science) disciplines [20, 19, 21–22]. The advantages of doing so are that researchers can produce a more comprehensive description of social phenomena. Additionally, formulating hypotheses about group differences in variance may allow researchers to test theories from different angles. Thus, investigating group differences in variance may be another piece of the puzzle when trying to understand why some ethnic or racial groups continue to exhibit large ethnic penalties while others are quicker to achieve structural parity [23, 4, 24, 13].

However, the idea of expanding the scope of inquiry beyond mean differences has remained on the scientific sideline; this methodology is rarely used in sociology and related disciplines (e.g., [21] in sociology, [25] and [26] in psychology or [27] in economics). We speculate that the primary reason that researchers do not use this methodology is because they are not used to thinking about the relationship between (a set of) explanatory factors and the difference in variance for an outcome variable. Additionally, researchers continue to think in terms of group mean differences because of the lack of theoretical models about variance differences, the lack of predictions about variance differences, and the lack of a conceptual foundation of distributional inequality.

The area of ethnic inequality may thus be exceptionally well-suited for research on distributional inequality. The area of ethnic inequality may thus be exceptionally well-suited for research on distributional inequality. We conceive of immigrant integration in terms of the eroding of distributional differences between majority and minority members, which occurs across generations [2, 28]. Based on this conceptualization, the process of immigrant integration is considered complete when distributional differences between have vanished immigrants and the majority population have vanished. At this point, intergroup inequality is expected to transition from vertical ethnic stratification toward ethnic equality or horizontal ethnic inequality. Furthermore, minority educational achievement is critical to the study of integration. In the short term, minority educational achievement serves as a yard stick for measuring educational attainment. In the long-term, minority educational achievement serves as a yardstick for measuring a host society’s labor market integration, or more generally, for structural integration [4, 24].

The recent abundance of comparative data has generated renewed interest in classic research questions in education research, namely, the relationship between cross-national differences in education system characteristics and cross-national variation in competency levels as well as ethnic competency penalties [29–37]. We aim to contribute to the literature on this topic by focusing on one specific area that lends itself to investigation within a distributional inequality framework: the efficiency-equality trade-off [38–40]. According to this classic hypothesis, education systems that separate students into a large number of tracks early on (based on student ability) achieve higher average competency (i.e., are more efficient) than comprehensive education systems; however, this efficiency comes at the expense of equality in student competency scores.

Hence, we take a tentative first step toward exploring the benefit of moving beyond mean differences by investigating cross-national ethnic penalties in math competencies. Our data come from the latest wave of the Program of International Student Assessment (PISA). The primary goal of the present study is to investigate whether declining mean ethnic penalties in educational achievement across generations are mirrored by an increase in ethnic mobility potentials. Deviating from much previous work in this field, this investigation will redirect attention to the conceptual foundations of research on immigrant integration—the gradual erosion of distributional differences—rather than examining mean differences between majority and minority populations. Based on general ideas derived from the literature on ethnic inequality in educational achievement, the second aim of this study is to examine whether the characteristics of education systems moderate the extent of distributional inequality between first- and second-generation immigrants relative to majority student populations. The empirical part of this study relies on a class of statistical models called mixed-effect location scale models. These models enable us to simultaneously estimate mean differences and (residual-)variance differences between immigrants and majority students by using a multilevel framework in which students are nested within schools and within countries [21]. In addition to investigating distributional inequality, we aim to remedy one of the major limitations of many studies in this field by examining immigrants’ educational achievement relative to the respective host country’s majority student population. This allows us to meaningfully assess the state of cross-national variation in ethnic stratification (e.g., [41,37].

### Education systems and ethnic inequality

#### General mechanisms

Education systems are typically classified according to two dimensions: standardization and stratification [42]. Standardization refers to the extent to which there are nationwide criteria for educational standards, such as having a system in place that holds schools accountable for their performance through teacher monitoring, regular inspections, and standardized exams [34]. Stratification, which is central to this study, is the extent to which an education system is stratified into distinct educational tracks. At the end of each track, students acquire different educational credentials. Students are placed in tracks based on their prior educational achievement; however, the age at which selection occurs and the number of distinct tracks can vary widely across countries. For example, Austria and Germany have highly stratified education systems, whereas Australia, Canada, and the Scandinavian countries are at the lower end of the stratification spectrum [42].

The way an education system is structured reflects a country’s underlying philosophy on education. Proponents of stratification argue that grouping students into tracks based on their educational achievement leads to a more homogenous student body with respect to students’ abilities, creating a supportive learning environment that allows the school to cater to the students’ specific educational needs [38,43–44]. There is some indication of differential competency growth rates across tracks; there are especially strong competency gains in the higher tracks. These strong gains for student in higher tracks raise the overall mean competency level in these education systems and increase the variance in the student population’s overall distribution of competency scores [45–46]. Critics have argued that stratification leads to a more homogenous student body with respect to both students’ abilities and students’ socioeconomic status [47]. Less talented students and students with less supportive learning environments at home may not get the benefit of interacting with more competent peers. These students may have lower self-esteem, because they attend schools that are perceived as having lower status [48–49]. Moreover, since students with lower socioeconomic status are less likely to be placed in higher tracks, they do not profit from the higher competency growth rates found in higher tracks; this exacerbates mean differences in competency across tracks and impacts the overall variance in competency. This is the primary focus of the literature on cross-national variation in the degree to which social origin correlates with educational outcomes (i.e., the social gradient), suggesting that the level of education system stratification increases the social gradient (e.g., [50–52]).

In sum, the empirical evidence is inconsistent and does not support one side over the other (cf. [47] and [53–54] for more general reviews). Additionally, existing research has focused on the efficiency aspect of stratification. Few, if any, studies have directly tackled the question of equality. Instead, researchers have provided indirect evidence about equality; for example, Hanushek and Wößmann [40] used a difference-in-difference design and country-level aggregate data to demonstrate that inequality increases after accounting for primary school inequality. Studies exploiting within-country variation in education systems and studies that provide (quasi-)experimental evidence based on policy changes tend to support the idea greater homogeneity in the student body is associated with higher efficiency, but the strength of the association is typically small [55–58].

#### Education systems and ethnic educational achievement

While the ethnoracial distinctions categorizing people as ‘immigrants’ often vary contingent on their sociocultural context [59], scholars widely agree that immigrant students (and, albeit to a lesser extent, second generation students) often have lower average scores on competency tests than do majority member students [60–61]. However, the gap between minority and majority students’ school achievements shows substantial cross-national and between group variation. In fact, a few ethnic groups score on par with or even outperform majority students [62,37]. A large body of national and cross-national research provides compelling evidence that ethnic penalties are strongly linked to primary effects of social origin (e.g., [5,62–65]. Primary effects posit that students from privileged socioeconomic backgrounds show higher school achievement than students with low socioeconomic status, because the parents in the privileged group may provide a more conducive learning environment at home and have more resources to compensate for any disadvantages. Many immigrants have a lower socioeconomic status than the majority population; this difference typically declines across generations. Hence, differences in socioeconomic status typically account for large parts of the educational achievement gap between minority and majority population students.

In line with previous research, we expect that minority students will have lower average scores on competency tests than majority students (H1a). The positive correlation between socioeconomic status and competence also has implications for variance in competence: Because the socioeconomic status distributions can differ substantially between majority and minority students, the majority student group will likely generate or cover a larger spectrum of possible competency scores. Conversely, minority students, who tend to come from low socioeconomic backgrounds, are on average less likely than their advantaged, majority peers to achieve high scores. Therefore, the average competency scores for minority students are more likely to be clustered at the lower end of the competency distribution. Moving beyond mean differences, we expect competency scores for minority students to be more compressed than the scores for majority students, conditional on the mean differences (H2a).

Critical features of education systems may moderate ethnic distributional differences. Early educational tracking may give disadvantaged students less time to catch up and ultimately demonstrate their academic abilities [5]. Conversely, tracking at older ages may give education systems more information regarding students’ scholastic abilities. The more information that education systems have about a student’s scholastic abilities, the better they will be at assessing the student’s likelihood of success at higher levels of education [66]. Minority students may end up in suboptimal tracks for a number of reasons. They may have less information on a host society’s education system, or they may be overwhelmed by the number of parallel tracks to choose from in the stratified systems. Additionally, since track selection is predominantly ability-based, minority students are more likely to be placed in lower tracks. Consequently, minority students do not profit from the steeper competency gains found in higher tracks. Because stratification strengthens the influence of socioeconomic status on educational trajectories, we expect that ethnic penalties in mean competency are higher, on average, in more stratified education systems (H1b). From a distributional perspective, the arguments discussed here also apply to ethnic mobility potentials. When the impact of socioeconomic background on competency is amplified in some education systems, minority students, relative to majority students, will cluster more frequently in lower tracks, will have less competency growth, and will have narrower distributions of competency scores. Therefore, conditional on mean differences, we expect the distribution of competency scores for minority students to be narrower than the distribution for majority students in more stratified education systems (H2b). A central tenet of research on immigrant integration is that the structural positions of immigrants and majority members tend to converge across generations [2,4,67–68].

Previous research has assessed the speed and the extent of generational convergence by comparing structural integration outcomes, such as average educational achievement and/or average labor market income differences. Notwithstanding differences across receiving contexts and origin group, second-generation immigrants have been shown to have higher achievement and better labor market integration than first-generation immigrants [53,67,5]. In particular, generational convergence in educational outcomes has been linked to the fact that second-generation immigrants face fewer language barriers and have more knowledge of a host country’s educational institutions, leading to higher achievement than first-generation immigrants [64,61]. What remains unclear is whether the decline in ethnic penalties for educational achievement across generations is associated with an increase in ethnic mobility potential. Thus, we hypothesize that for second-generation minority students, relative to first-generation minority students, ethnic mean penalties will be smaller (H3a), and ethnic mobility potentials will be higher (H3b).

### Data and methods

#### Data

To study inequality in ethnic achievement, we relied on data from the 2012 PISA, which covered the largest number of countries to date [69]. In line with existing research on achievement differences, competency in mathematics was measured as the mean of five plausible values published by the OECD [62,70,37]. Examining math competency, instead of the commonly used reading scores, minimizes the risk of language dependency bias [35]. The sample was restricted to students at age 15 with two majority parents (i.e., majority students) or students with at least one foreign-born parent (i.e., minority students). Minority students born outside of the host country were classified as first-generation immigrants only if they entered the host country before the age of 10; this ensures sufficient exposure to the host country’s education system. Minority students born in the host country were considered second-generation minorities. In total, the analysis sample contained 368,481 students, 14,938 schools, and 61 countries. (No data on education systems in Albania and Serbia were available. Additionally, we combined the data from Perm with the Russian data, and we combined the data from Taipei with the Chinese data). Individual participation in PISA is voluntary and fully anonymized, and all respondents have agreed that their information may be used for scientific research without their further consent. We therefore did not seek formal approval of this study by an ethics committee before undertaking this research. Note that although the PISA data is unrivaled in its scope of countries cases, it is, like other cross-national data sets on student competencies, unable to provide a direct test of the efficiency-equality trade-off hypothesis because of its lack of measures for early school career abilities or more general measures of cognitive abilities. However, it is nonetheless an essential data source for exploring the impact of education systems characteristics in the context of ethnic achievement inequality, since we are primarily interested in their *moderating* influence.

To account for compositional differences in socioeconomic status across countries and, more importantly, socioeconomic differences between majority and minority students, we included a range of student information that determines socioeconomic status (e.g., PISA’s weighted index of parental education, job status, occupational status, and home possessions) and the availability of educational resources at home (e.g., PISA’s weighted index of students having access to a study desk, a quiet place to study, a computer to do schoolwork, educational software, a personal calculator, books to help with school work, and dictionaries). We also collected information on students’ gender (0 = male, 1 = female), school grade, and number of parents in the household. Because detailed information on country of origin or parents’ country of origin is not always available for minority students, we instead used region of origin (0 = majority student, 1 = Africa, 2 = Europe, 3 = Asia, 4 = other.

We examined school-level information to control for changes in student body composition due to cross-national differences in education systems. Socioeconomic school composition is assessed by using the proportion of minority students, the highest average parental occupational status at the school, and the associated standard deviation. This latter measure aims to account for school homogeneity in terms of socioeconomic status. The homogeneity of the student body was further assessed by measuring the extent to which teachers reported being hindered by students disrupting lessons and the extent to which they reported having to teach students with different learning abilities in the same class (1 = not at all, 4 = a lot). We also included information on school resources, such as student-teacher ratio, teacher shortage (i.e., a weighted index recording the shortage of qualified science, mathematics, host country’s language teachers, and other subject teachers), quality of educational resources (i.e., a weighted index recording the shortage of science equipment, instructional material, computers, Internet, software, and library material), and whether the school was private or public.

To measure differences in education system stratification, we relied on aggregated PISA data and auxiliary data from UNESCO’s World Data on Education [71]. Specifically, we used information on age when track selection takes place and number of parallel tracks at age 15. To assess the extent of ability grouping, we measured the proportion of schools in a country that grant students admission based solely on their prior academic record. To control for the possibility that de facto tracking is present in even the most comprehensive education systems (due to residential segregation, for instance), we included information on the ‘relevance’ of schools. Relevance is measured as the within-country intra-class correlation of the school level [57]. This measure can be interpreted as the relative proportion of a country’s variance in math competency that can be attributed to school differences. Even in countries with comprehensive school systems and low levels of residential segregation (e.g., Finland, Norway, Sweden), between 10% and 20% of the variance in math competency can be attributed to school differences. For countries with comprehensive school systems and moderate levels of residential segregation (e.g., Australia, Canada), between 20% and 30% of the variance in math competency can be attributed to school differences. Additionally, we controlled for educational spending (measured as a percentage of total government expenditure [72]) and income inequality (Gini index) [73] in 2005.

Table 1 shows descriptive statistics for all independent variables.

**Table 1:** Descriptive statistics for independent variables (N_country_ = 61, N_school_ = 14,938, N_students_ = 368,481).

	Range	Mean/ Proportion	SD
Individual level			
Majority student	0–1	0.95	
First-generation minority student	0–1	0.02	
Second-generation minority student	0–1	0.03	
Socioeconomic status	-5.95–3.69	-0.29	1.14
Home educational resources	-3.93–1.12	-0.20	1.07
Male (ref.: female)	0–1	0.49	
Grade	7–12	9.66	0.74
Single parent (ref.: both parents present)	0–1	0.12	
Region of origin (ref: majority student)			
Africa	0–1	0.01	
Europe	0–1	0.02	
Asia	0–1	0.02	
Other	0–1	0.02	
School level			
Proportion of minority students	0–1	0.06	0.14
Avg. parental highest occupational status	11.01–84.08	47.50	12.99
Sd^a^(avg. parental highest occupational status)	0–41.39	18.14	4.46
Frequency of student disruptions	1–4	2.18	0.78
Abilities within class too heterogeneous	1–4	2.48	0.88
Student-teacher ratio	0.07–1018	15.19	14.84
Teacher shortage	-1.09–3.60	0.03	1.05
Quality of educational resources	-3.59–1.98	-0.12	1.11
Private school (ref.: public school)	0–1	0.19	
Country level			
Age at selection	7–17	13.98	2.01
Number of tracks	1–5	2.59	1.20
Ability-based admission	0–1	0.42	0.25
ICC^b^ school level	0.13–0.70	0.42	0.15
Educational expenditure	2.92–7.66	4.61	1.06
Gini	25.59–53.54	35.67	6.04

^a^ Sd refers to the standard deviation of the average parental highest occupational status at the school level.

^b^ ICC refers the intraclass correlation of math scores at the school level.

#### Methods

We modeled distributional inequality with one type of mixed-effects location scale model [21] (see [20,25,22] for similar conceptual frameworks but different implementations). This type of statistical model was first developed as a diagnostic tool to detect heteroscedasticity [74]; however, researchers have recently acknowledged that the model can be used to investigate inequality. Location scale models estimate the relationship between independent variables and the mean of the dependent variable (i.e., the location) as well as the relationship between independent variables and the error variance (i.e., the scale). We illustrate the underlying idea using the following linear model with random intercepts and random slopes at levels 2 and 3 (individuals *i* nested within level 2 units *j* and level 3 units *k*):

$$\begin{array}{l} Level\;1:y_{ijk}∼N(\mu_{ijk},\sigma_{\varepsilon }^{2})for\;i=1,\ldots \;N;j=1,\ldots ,J\;and\;k=1,\ldots ,K\\ \mu_{ijk}=\beta_{0jk}+\beta_{1jk}X+\varepsilon_{ijk}\\ Level\;2:\;\beta_{0jk}+\beta_{01}Z+u_{0jk}\\ \beta_{1jk}=\beta_{10k}+u_{1jk}\\ Level\;3:\;\beta_{00k}=\gamma_{000}+\gamma_{001}W+u_{00k}\\ \beta_{1jk}=\gamma_{100}+\gamma_{101}W+u_{10k} \end{array}$$ Location model (Eq 1)

y_ijk_ is a normally distributed response vector with mean μ_ijk_ and variance σ_ε_^2^. On level 1, the mean (μ_ijk_) is written as a function of the random intercept (β_0jk_), a level 1 predictor (X), the predictor’s random coefficient (β_1jk_), and the level 1 residual error term (ε_ijk_). On level 2, β_0jk_ is the random intercept that is defined by its own random intercept (β_00k_), a level 2 predictor (Z), the predictor’s corresponding coefficient (β_01_), and the level 2 residual error term (u_0jk_). β_1jk_ is defined as a function of the level 2 effect for the slope (β_10k_), and variance around this value is captured by u_1jk_. Level 3 comprises the grand mean (γ_000_), a level 3 predictor (W), the predictor’s associated coefficient (γ_001_), and a residual error term (u_00k_). β_1jk_ denotes the random slope at level 3. The level 3 model also includes the slope’s mean (γ_100_), a cross-level interaction (γ_001_) with level 3 predictor (W), and the variance around the average slope (u_10k_). The residual error terms (ε_ijk_, u_0jk_ and u_00k_) are assumed to have a mean of zero and variances of σ^2^_εijk_, σ^2^_u0jk_ and σ^2^_u00k_, respectively. In this study, the model described in Eq 1 had students nested within schools nested within countries. The predictor (X) represented the differences between majority and minority students’ math scores. This difference was allowed to vary across schools and countries; this difference also varied as a function of country characteristics (W).

Instead of making assumptions about the error term on level 1, location scale models explicitly define it as a function of explanatory variables. Thus, we adapted Eq 1 to include the following random intercept and random slope scale model (individuals *i* nested within level 2 units *k*):

$$\begin{array}{l} Level\;1:\log\left(\sigma_{\varepsilon ik}^{2}\right)=\lambda_{0k}+\lambda_{1k}X\;for\;i=1,\ldots ,N\;and\;k=1,\ldots ,K\\ Level\;2:\lambda_{0k}=v_{00}+v_{01}W+v_{0k}\\ \lambda_{1k}=v_{10}+v_{11}W+v_{1ik} \end{array}$$ Scale model (Eq 2)

Compared to Eq 1, the variance (σ^2^_εik_) in Eq 2 was allowed to vary across level 1 and level 2 units, as indicated by the *i* and *k* subscripts. Note that level 2 referred to countries rather than schools. Moreover, σ^2^_εik_ was assumed to have a log-normal distribution to ensure that the variance was positive. On level 1, the variance was modeled as a function of a random intercept (λ_0k_), the level 1 predictor (X), and the predictor’s associated random slope (λ_1k_). The intercept (λ_0k_) was allowed to vary across level 2 units and was defined by the grand mean (ν_00_), the level 2 predictor (W), the predictor’s corresponding coefficient (ν_01_), and specific level-2 variance (v_0k_). The random slope (λ_1k_) was defined by a mean slope (ν_10_), a cross-level interaction (ν_11_), the level 2 predictor (W), and the variance around the average slope (v_1k_). In substantive terms, the scale part of Eq 2 estimated the difference between majority and minority students’ variance in math scores, with students nested hierarchically within countries. Similar to Eq 1, this difference was allowed to vary across countries; the difference also varied as a function of country characteristics (W). Regression weights for the scale equations (λ and ν) can be interpreted as the (average) difference in (log-transformed) variance associated with a one-unit change in the independent variables. Hence, the interpretation of the regression weights is similar to that of coefficients from logistic regression models. In logistic regression models, the exponentiated coefficients refer to odds ratios; in the current model, the exponentiated coefficients referred to the factor change in the variance. Estimation was based on a two-stage process. In the first stage, the mean model was estimated. In this study, we relied on three-level models with random intercepts and random slopes in which students were hierarchically nested within schools and countries. Additionally, ethnic penalties were allowed to vary across countries and schools in the current model. In the second stage of estimation, the squared residuals of the first-stage estimation now became the dependent variable. The dependent variable’s relationship with the independent variables was then estimated using gamma regression models (with log link function). Since we were primarily interested in cross-national variance, we estimated two-level models with random intercepts and random slopes in which students were hierarchically nested within countries and in which ethnic mobility was allowed to vary across countries. All models were based on maximum likelihood estimation using the lme4-package for R [75–76; see [25] for a Bayesian version).

## Results

Because previous studies have used PISA data to examine cross-national variance in mean achievement differences (see [37]), this study focused on multivariate findings. In this results section, we start by presenting a baseline model that only includes the difference between majority and minority students’ math scores and the corresponding random slopes, thereby giving insight into ‘gross’ ethnic penalties. The school level and the country level contributed an approximately equal amount of variance in average math competency scores: 28% [2830.9/(4293.1+2830.9+2940.5)] of the variance could be attributed to the school level, and 29% of the variance could be attributed to the country level. Scaling the competency measures in the PISA data makes it easier to interpret the substantive significance of these results. The competency measures had a sample mean of 500 points and a standard deviation of 100 points. Therefore, differences in mean PISA scores can be interpreted in terms of percentage points of a standard deviation. According to mean coefficients β of Model 0 (presented in Table 2), ethnic penalties were highest for first-generation immigrants: They scored an average of 42 points lower than majority students. Second-generation immigrants scored 32 points lower than majority students. The random slopes suggest that these averages ‘hide’ substantial cross-national variance in ethnic penalties. The 95% confidence interval of the slopes for first-generation immigrants revealed that ethnic penalties could be over 105 points in some countries (approximately one standard deviation of PISA’s math competency measurement); however, some immigrants outperformed majority students by 22 points in other countries (-41.61±1.96*32.38 = [-105.07; 21.85]). Similarly, ethnic penalties for second-generation immigrants varied cross-nationally, albeit to a lesser degree. The estimated confidence interval of the slopes for second-generation immigrants was [-84.25; 20.11]. Unsurprisingly, this first model replicated findings from the literature and lent support for H1a and H3a.

**Table 2:** Mixed-effects location scale model of math competence, PISA 2012; N_country_ = 61, N_school_ = 14,938, N_students_ = 368,481. (β-coefficients based on linear regression, λ-coefficients based on gamma regression, standard errors in parentheses).

	M0		M1 = M0 + individual characteristics	
	β	λ	β	λ
Intercept	471.78* (6.97)	8.27* (0.06)	479.26* (6.87)	8.15* (0.06)
Majority student (ref.)				
First-generation minority student	-41.61* (6.89)	-0.26* (0.08)	-30.29* (6.31)	-0.14* (0.07)
Second-generation minority student	-32.07* (5.72)	-0.15* (0.07)	-26.72* (4.93)	-0.17* (0.08)
Age at selection ^ƚ^				
Number of tracks ^ƚ^				
Ability-based admission ^ƚ^				
First-generation minority student*Age at selection				
Second-generation minority student*Age at selection				
First-generation minority student*Number of tracks				
Second-generation minority student*Number of tracks				
First-generation minority student* Ability-based admission				
Second-generation minority student* Ability-based admission				
Socioeconomic status			11.96* (0.14)	
Home educational resources^+^			4.55* (0.12)	
Male (ref.: female) ^+^			17.33* (0.22)	
Grade^+^			36.85* (0.23)	
Single parent (ref.: both parents present) ^+^			2.82* (0.32)	
Region of origin (ref: majority student)				
Africa	-13.45* (5.51)	0.04 (0.10)	-8.32 (5.13)	0.00 (0.10)
Europe	26.07* (4.15)	0.09 (0.07)	21.81* (3.83)	0.10 (0.07)
Asia	35.48* (4.27)	0.04 (0.07)	36.00* (3.95)	0.08 (0.07)
Other	7.18 (4.63)	0.04 (0.08)	13.22* (4.28)	0.03 (0.08)
Proportion of minority students^+^				
Avg. parental highest occupational status^ƚ^				
Sd^a^(avg. parental highest occupational status) ^ƚ^				
Frequency of student disruptions^+^				
Abilities within class too heterogeneous^+^				
Student-teacher ratio^ƚ^				
Teacher shortage^ƚ^				
Quality of educational resources^ƚ^				
Private school (ref.: public school) ^+^				
ICC^b^ school level^ƚ^				
Educational expenditure^ƚ^				
Gini^ƚ^				
Variance terms				
Var(Country)	2,940.5	0.22	2,858.9	0.22
Var(School)	2,830.9		1,958.1	
Var(Student)	4,293.1	1.99	3,816.6	2.00
Slopes				
Country(first-generation minority students)	32.38	0.29	28.17	0.20
Country(second-generation minority students)	26.62	0.12	22.47	0.12
School(first-generation minority students)	32.59		28.37	
School(second-generation minority students)	14.25		11.94	
BIC^c^	4,126,694	6,619,336	4,080,395	6,534,512
AIC^d^	4,126,478	6,619,185	4,080,125	6,534,361

* p < .05; + grand-mean centered variable

ƚ = standardized variable.

^a^ Sd refers to the standard deviation of the average parental highest occupational status at the school level.

^b^ ICC refers the intraclass correlation of math scores at the school level.

^c^ BIC denotes the Bayesian Information Criterion.

^d^ AIC denotes the Akaike Information Criterion.

The findings presented in the variance coefficients column λ of Model 0 suggest that approximately 10% [0.22/(0.22+1.99)] of variance differences was due to cross-country variation. As predicted in H1b, both first- and second-generation immigrants were more concentrated at the lower end of the distribution compared to majority students. As predicted in H3b, we observed the standard pattern of generational differences in variance: The distribution of competency scores for first-generation immigrants’ was narrower than the distribution for second-generation immigrants. More specifically, math scores for first-generation students varied by a factor of 0.77 (e^-0.26^), and scores for second-generation students varied by a factor of 0.86 (e^-0.15^), where a factor of 1 would suggest no variance difference compared to majority student scores. Similar to the ethnic penalties, ethnic mobility also varied cross-nationally. The corresponding 95% confidence intervals for first-generation and second-generation students were [-0.83; 0.31] and [-0.39; 0.09], respectively. In extreme cases, the variance in math competency was smaller by a factor of 0.44, but there were also countries where the variance in math competency was higher for immigrants than for majority students.

**Fig 1:**
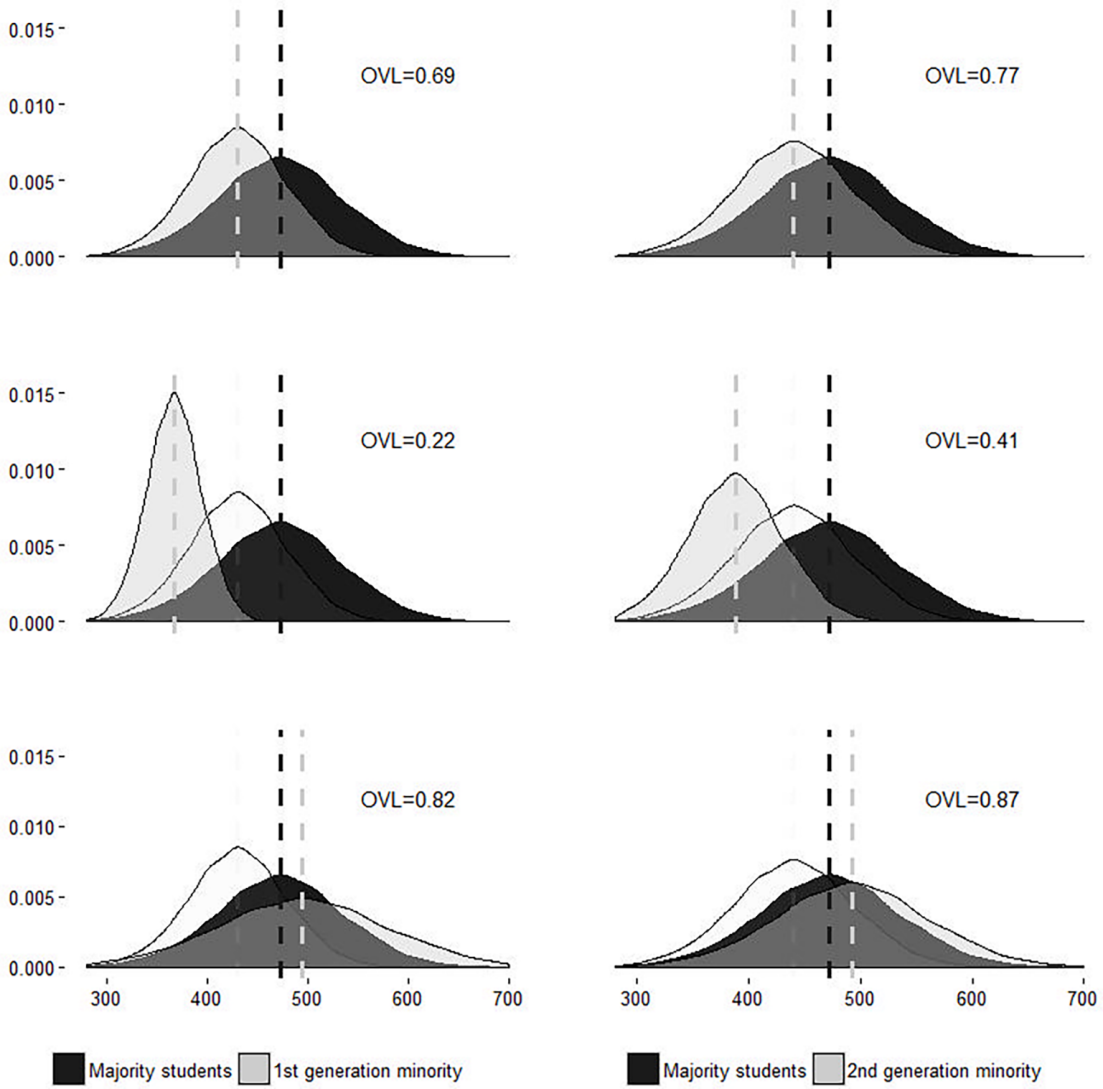
Simulation of distributional inequality between first- and second-generation minority students and majority students. OVL refers to the overlapping coefficient values which range from 0 to 1 and indicated the percentage overlap in the depicted distributions.

To provide a more intuitive picture of these gross ethnic mean penalties and the ethnic mobility potentials, we simulated data based on the Model 0 coefficients. Fig 1 depicts the corresponding distributions, in which means are represented by dashed lines. In each panel’s top right corner, the overlapping coefficient values (OVL) are shown. OVL values have a theoretical range of 0 to 1 and can be interpreted as the percentage overlap between the majority and minority distributions [77]. The top panels show the ‘average’ scenario and neatly illustrate generational differences. These panels show that first-generation minority students had lower math scores and were more tightly clustered within their distribution, compared to second-generation and majority students (top right panel). Overall, the math competency distributions still showed a comparatively large degree of overlap for first- and second-generation students (OVLs = 0.69 and 0.77, respectively). The middle panels are based on lower bounds of the 95% confidence intervals of the random slopes. These lower bounds represented the scenario for the ‘most disadvantaged’ students (for reference, the average scenario distribution is shown in the background). Visually, the ethnic mean penalties appeared to be more severe and the ethnic mobility potential appeared to be significantly restricted; this is indicated by the clustering of math scores around the minority group means. This was also evidenced by the reduced overlap in minority and majority distributions (OVLs = 0.22 and 0.41, respectively). Finally, the scenario for the ‘least disadvantaged’ students (see the bottom of Fig 1), which showed a minority advantage, found minority students scoring higher, on average, and having higher variance in math scores. Out of all three scenarios, the latter scenario depicted the lowest levels of group inequality with strongly overlapping competence distributions, especially for second-generation minority students (OVLs = 0.82 and 0.87, respectively).

The subsequent models added student background characteristics (M1), resulting in the greatest decline in majority-minority differences across all estimated models. Accounting for compositional differences in the home environment by controlling for socioeconomic status, home educational resources and single-parent household (as well as student sex and grade) reduced the ethnic mean penalties for first-generation minority students by approximately 10 points (to 30) and reduced the penalty by approximately 5 points for second-generation minority students. These compositional differences also accounted for a substantial part of the restricted ethnic mobility that first-generation students face; the compositional differences increased ethnic mobility potentials by 13 percent from -0.26 to -0.14 (1-(e^-.14^/e^-.26^)). For second-generation students, ethnic mobility potentials only declined slightly after accounting for home environment (from -0.15 to -0.17). Finally, differences in home environment explained some of the cross-national variance in ethnic penalties and ethnic mobility potential, as evidenced by the decreasing magnitude of the random slopes (the ethnic penalties for first- and second-generation students decreased by 13% to 16%; the ethnic mobility potentials of first-generation students decreased by one-third). Differences in home environment did not explain cross-national variance in ethnic mobility potentials for second-generation minority students.

Model 2 in Table 3 also included country characteristics in the form of our three key indicators of education system differences as well as a number of controls in order to investigate their relationship with differences in average competency and competency spread for all students irrespective of majority or minority status. With respect to both mean differences and variance differences for math scores, we did not find any association between age at track selection and number of tracks. We did find that a higher proportion of schools practicing merit-based admission was positively associated with both country-level competency and the variance in competency. When comparing a country with an average proportion of merit-based admission schools (about 42%; see Table 1) with a country where 67% of schools (one standard deviation above the mean) practice merit-based admission, the average math score of the latter country was 24 points higher than the former. Similarly, when comparing the variance in competence between the two countries, the variance of the country with more merit-based schools was higher by a factor of 1.09. Although this finding is in line with the general notion of the efficiency-equality trade-off, the null findings for age at selection and number of parallel tracks therefore provided only limited support for this hypothesis in the general student population. Moreover, we caution readers again that PISA data is only of limited usefulness for a direct test of the efficiency-equality trade-off.

**Table 3:** Mixed-effects location scale model of math competence, PISA 2012; N_country_ = 61, N_school_ = 14,938, N_students_ = 368,481. (β-coefficients based on linear regression, λ-coefficients based on gamma regression, standard errors in parentheses).

	M2 = M1+country characteristics		M3 = M2+cross-level interactions	
	β	λ	β	λ
Intercept	471.43* (6.00)	8.13* (0.04)	471.01* (6.99)	8.13* (0.04)
Majority student (ref.)				
First-generation minority student	-29.92* (6.31)	-0.16* (0.08)	-29.58* (6.10)	-0.16* (0.08)
Second-generation minority student	-27.35* (4.97)	-0.14* (0.06)	-26.86* (4.83)	-0.13* (0.06)
Age at selection^ƚ^	-9.95 (6.16)	-0.02 (0.04)	-8.64 (6.73)	-0.02 (0.05)
Number of tracks^ƚ^	1.83 (6.51)	0.00 (0.050)	5.32 (7.22)	-0.00 (0.05)
Ability-based admission^ƚ^	24.31* (5.33)	0.09* (0.04)	21.86* (5.96)	0.09* (0.04)
First-generation minority student*Age at selection			-3.77 (6.51)	0.13* (0.06)
Second-generation minority student*Age at selection			-2.01 (4.43)	0.05* (0.01)
First-generation minority student*Number of tracks			-9.68 (7.03)	0.09 (0.08)
Second-generation minority student*Number of tracks			-7.89 (5.00)	0.03 (0.04)
First-generation minority student* Ability-based admission			11.23 (7.48)	0.02 (0.05)
Second-generation minority student* Ability-based admission			6.96 (3.87)	0.00 (0.03)
Socio-economic status	11.96* (0.14)		11.96* (0.14)	
Home educational resources^+^	4.55* (0.12)		4.55* (0.12)	
Male (ref.: female) ^+^	17.33* (0.22)		17.33* (0.22)	
Grade^+^	36.85* (0.23)		36.85* (0.23)	
Single parent (ref.: both parents present) ^+^	2.82* (0.32)		2.82* (0.32)	
Region of origin (ref: majority student)				
Africa	-8.66 (5.16)	-0.00 (0.10)	-9.20 (5.17)	-0.03 (0.10)
Europe	21.36* (3.89)	0.10 (0.07)	20.98* (3.9)	0.07 (0.07)
Asia	35.51* (4.00)	0.07 (0.07)	34.83* (4.02)	0.04 (0.07)
Other	12.95* (4.34)	0.03 (0.08)	12.48* (4.35)	0.01 (0.08)
Proportion of minority students^+^				
Avg. parental highest occupational status^ƚ^				
Sd^a^(avg. parental highest occupational status) ^ƚ^				
Frequency of student disruptions^+^				
Abilities within class too heterogeneous^+^				
Student-teacher ratio^ƚ^				
Teacher shortage^ƚ^				
Quality of educational resources^ƚ^				
Private school (ref.: public school) ^+^				
ICC^b^ school level^ƚ^	-28.99* (6.07)	-0.31* (0.05)	-28.36* (6.84)	-0.31* (0.05)
Educational expenditure^ƚ^	-3.60 (7.05)	-0.00 (0.05)	-3.49 (7.10)	-0.00 (0.05)
Gini^ƚ^	-15.32* (5.71)	-0.08* (0.04)	-16.56* (5.77)	-0.08* (0.04)
Variance terms				
Var(Country)	1,733.1	0.08	1,709.9	0.08
Var(School)	1,958.2		1,958.2	
Var(Student)	3,816.6	2.00	3,816.6	2.00
Slopes				
Country(first-generation minority students)	28.00	0.20	25.27	0.18
Country(second-generation minority students)	22.22	0.12	20.29	0.10
School(first-generation minority students)	28.38		28.39	
School(second-generation minority students)	11.94		11.94	
BIC^c^	4,080,443	6,534,613	4,080,514	6,533,676
AIC^d^	4,080,108	6,534,396	4,080,114	6,534,395

* = p < .05; + = grand-mean centered variable

ƚ = standardized variable.

^a^ Sd refers to the standard deviation of the average parental highest occupational status at the school level.

^b^ ICC refers the intraclass correlation of math scores at the school level.

^c^ BIC denotes the Bayesian Information Criterion.

^d^ AIC denotes the Akaike Information Criterion.

Model 3 suggested that the three education system characteristics were also not associated with cross-national variance in ethnic mean penalties—that is, there were no statistically significant cross-level interactions to support hypothesis 2a. However, the λ column of Model 3 indicated that the age at track selection was positively associated with ethnic mobility potentials. In countries where the age at track selection is one standard deviation higher (i.e., selection takes place ~ 2 years later in a student’s school life), ethnic mobility potential for first-generation students was nearly equal to that of majority students (-0.16+0.13 = -0.03, which corresponds to a factor of 0.97) and only moderately lower for second-generation students (-0.13+0.05 = -0.08). This finding is in line with hypothesis 3b: Given their lower average competence scores, immigrants were more likely to be in lower tracks when they had less time to demonstrate their academic ability and less time to profit from interaction with their peers. This resulted in a narrow distribution of competency in which scores were clustered at the lower end.

The relationship between age at track selection and ethnic mobility potentials remained robust when also including the school-level explanatory variables presented in Model 4 (see Table 4). The differences between stratified and comprehensive school systems were due to the fact that stratified systems lead to changes in the student body composition by separating students into different schools and tracks. There is evidence that homogenous student bodies lead to gains in efficiency: average math scores are higher in schools with more homogeneity in socioeconomic status (as measured by school-level standard deviation of highest parental occupational status) and schools where teachers less often voice concerns that class ability levels are too heterogeneous. Moreover, average math scores are lower in schools with a higher frequency of student disruptions. However, the claims that homogeneity leads to higher competency assumes that student disruptions are systematically due to students being unable to keep up with the pace of the course or, conversely, feeling bored because the pace is too slow. Unfortunately, we are unable to assess the validity of this assumption.

**Table 4:** Mixed-effects location scale model of math competence, PISA 2012; N_country_ = 61, N_school_ = 14,938, N_students_ = 368,481. (β-coefficients based on linear regression, λ-coefficients based on gamma regression, standard errors in parentheses).

	M4 = M3 + school characteristics	
	β	λ
Intercept	474.05* (6.77)	8.13* (0.04)
Majority student (ref.)		
First-generation minority student	-29.63* (6.02)	-0.11* (0.07)
Second-generation minority student	-27.12* (4.70)	-0.11* (0.06)
Age at selection^ƚ^	-9.84 (6.41)	-0.03 (0.05)
Number of tracks^ƚ^	6.06 (8.09)	-0.00 (0.05)
Ability-based admission^ƚ^	18.81* (6.67)	0.10* (0.04)
First-generation minority student*Age at selection	-2.92 (6.44)	0.13* (0.06)
Second-generation minority student*Age at selection	-1.20 (4.28)	0.07* (0.03)
First-generation minority student*Number of tracks	-8.68 (6.96)	0.13 (0.09)
Second-generation minority student*Number of tracks	-6.75 (4.84)	0.05 (0.03)
First-generation minority student* Ability-based admission	10.35 (5.40)	0.01 (0.04)
Second-generation minority student* Ability-based admission	6.73 (3.72)	0.01 (0.02)
Socio-economic status	10.49* (0.14)	
Home educational resources^+^	4.64* (0.12)	
Male (ref.: female) ^+^	17.47* (0.22)	
Grade^+^	35.76* (0.22)	
Single parent (ref.: both parents present) ^+^	2.56* (0.32)	
Region of origin (ref: majority student)		
Africa	-10.08 (5.12)	-0.07 (0.09)
Europe	19.83* (3.84)	0.04 (0.06)
Asia	34.07* (3.96)	-0.01 (0.06)
Other	11.11* (4.27)	0.06 (0.07)
Proportion of minority students^+^	9.42* (3.16)	
Avg. parental highest occupational status^ƚ^	30.69* (0.42)	
Sd^a^(avg. parental highest occupational status) ^ƚ^	-3.62* (0.31)	
Frequency of student disruptions^+^	-8.34* (0.47)	
Abilities within class too heterogeneous^+^	-1.33* (0.40)	
Student-teacher ratio^ƚ^	-0.54 (4.27)	
Teacher shortage^ƚ^	0.29 (0.38)	
Quality of educational resources^ƚ^	0.17 (0.37)	
Private school (ref.: public school) ^+^	-10.15* (1.01)	
ICC^b^ school level^ƚ^	-22.01* (7.60)	-0.33* (0.05)
Educational expenditure^ƚ^	-5.28 (7.86)	-0.03 (0.05)
Gini^ƚ^	-9.88 (6.41)	-0.11* (0.04)
Variance terms		
Var(Country)	2,157.7	0.08
Var(School)	1,255.8	
Var(Student)	3,817.2	2.00
Slopes		
Country(first-generation minority students)	25.08	0.06
Country(second-generation minority students)	19.75	0.06
School(first-generation minority students)	27.84	
School(second-generation minority students)	12.33	
BIC^c^	4,074,922	6,535,085
AIC^d^	4,074,425	6,534,804

* = p < .05; + = grand-mean centered variable

ƚ = standardized variable.

^a^ Sd refers to the standard deviation of the average parental highest occupational status at the school level.

^b^ ICC refers the intraclass correlation of math scores at the school level.

^c^ BIC denotes the Bayesian Information Criterion.

^d^ AIC denotes the Akaike Information Criterion.

The R^2^ for the various random effects suggested that there was still a substantial amount of cross-national variance in average math scores that is unaccounted for (R^2^~0.27 [(2940.5–2157.7-)/2940.5]). Similarly, approximately 23% of the cross-national variance in ethnic mean penalties for first-generation minority students and 26% of the variance in ethnic mean penalties for second-generation minority students was explained. The final column of Model 4 (see Table 4) indicates that a larger part of cross-national variance in math competency variance was accounted for (R^2^~0.27 [(0.22–0.08)/0.22]). Approximately 70% [(0.29–0.06)/0.29] and 50% of cross-national variance in ethnic mobility potentials for first- and second-generation minority students, respectively, was accounted for. Importantly, cross-national variance in ethnic mobility potentials declined sharply when the mean model was adjusted for school-level differences. This reflected of the impact of education systems on ethnic mobility and suggested substantial within-country variance. It is also important to note that once all adjustments were made, the confidence intervals for the random slope of ethnic mobility potentials barely crossed over into the positive. This suggested that once compositional differences across groups and schools were accounted for, minority students had a narrower competence distribution than majority students.

## Summary and conclusions

In this study, we aim to redirect scholarly attention to immigrant integration as a process of eroding distributional inequality between the majority and minority groups over time and generations. On a methodological level, we introduce the use of mixed-effect location scale models to describe and explain such distributional inequality between groups. To this end, we move away from the prevailing focus on mean differences and acknowledge that variance differences can be substantive and insightful. Focusing on ethnic inequality in educational achievement as a core dimension of integration, we examine two basic cross-national comparative research questions. First, we ask whether declining ethnic inequality in competence (i.e., ethnic mean penalties) across generations is mirrored by an increase in ethnic mobility potentials (i.e., between-group differences in competency variance). Secondly, based on existing research on ethnic inequality in educational achievement, we ask whether cross-national differences in the structure of education systems affect ethnic penalties and ethnic mobility potentials.

We used both math competency data from the 2012 PISA and mixed-effects location scale models. Our results suggest that on average, minority students have lower competency scores. Moreover, minority students have less variance in competency; their scores are tightly clustered around the lower end of the distribution. We also find support for the notion that distributional inequality erodes over generations; second-generation minority students have lower ethnic penalties and higher ethnic mobility than their first-generation counterparts. We do not find compelling evidence that stratified education systems amplify ethnic mean penalties; the ethnic inequality in math competence is not larger in countries with early educational tracking. Most existing studies would have stopped their inquiries at this point. However, our investigation of cross-national differences in math competency *variance* generates important insights: Track selection at an older age is associated with higher ethnic mobility potentials. This indicates that the distribution of competency scores for minority students is wider, suggesting that minority students may benefit from more shared time with majority students. Thus, the empirical strategy outlined here might provide a constructive framework for future research investigating the effectiveness of interventions targeted at closing performance gaps between minority and majority members in educational settings.

There are a number of important extensions to this study that would tackle some of its limitations. For example, due to limitations in the data available we were unable to examine the role of anti-minority prejudice for affecting the difference between minority and majority students’ average school performance [78] and its variability. Consequently, filling this research gap remains as a logical next step for future research. Relatedly, the large scope of PISA data comes at the cost of details that it hides. Thus, we can only speculate about the plethora of group differences that are obscured in the distributions when the categories are as generic as ‘first-generation minority students’ and ‘second-generation minority students.’ Data permitting, more comprehensive insights could be expect from research designs that allow for a more detailed differentiation between immigrant students in terms of their origin countries. Still, the remarkable variance in the random slopes for ethnic mean penalties and ethnic mobility potentials suggest that differences between origin groups are a highly promising avenue for future research. The pace of generational advancement may be strongly associated with between-group differences in ethnic mobility potentials. This may explain how different groups can start with similar conditions and compositions but the disadvantaged socioeconomic positioning of one group remains or even deteriorates whereas another group might secure a socioeconomic positioning at a similar level as the majority. Related to this, origin groups that have very similar developmental trajectories (assessed by comparing their average integration outcomes) may differ substantially in their ethnic mobility potentials and, therefore, show distinct levels of socioeconomic achievement in the third generation and beyond. Because distributional inequality is fundamentally a conceptual framework, there is no limitation to the type of (ethnic) inequality that can be studied. Any type of research design can be used to study distributional inequality. There are a number of theories and hypotheses that already speculate about between-group differences in variance. Literature from other disciplines can enrich our understanding of distributional inequality, especially with respect to variance. For example, Braumoeller [18] created a conceptual framework that extended treatment effects in the mean to include the variance. Braumoeller also discussed various generic causal mechanisms and their association with changes in variance. To enhance our understanding of social phenomena and to improve our ability to formulate and test theoretical ideas about those phenomena, researchers should incorporate these ideas into the way that we conduct studies and think about intergroup inequality.
